# Antioxidant dressing therapy versus standard wound care in chronic wounds (the REOX study): study protocol for a randomized controlled trial

**DOI:** 10.1186/s13063-020-04445-5

**Published:** 2020-06-08

**Authors:** Inés María Comino-Sanz, María Dolores López-Franco, Begoña Castro, Pedro Luis Pancorbo-Hidalgo

**Affiliations:** 1 Department of Nursing, Faculty of Health Sciences, University of Jaén, Las Lagunillas S/N, 23071 Jaén, Spain; 2 Histocell S.L., Bizkaia Science and Technology Park, Derio, Bizkaia Spain

**Keywords:** Chronic wound, Wound healing, Hard-to-heal wounds, Antioxidant dressing, Oxidative stress

## Abstract

**Background:**

A wound that does not heal in the orderly stages of the healing process or does not heal within 3 months is considered a chronic wound. Wound healing is impaired when the wound remains in the inflammatory stage for too long. A range of factors can delay the healing process: imbalance between proteases and protease inhibitors in the wound bed; bacterial colonization and the presence of biofilm; and oxidative stress. Recently, wound management has improved significantly. A new antioxidant dressing has been developed, which combines an absorbent matrix obtained from locust bean gum galactomannan and a hydration solution with curcumin and N-acetylcysteine. This dressing combines the advantages of moist healing in exudate management and free radical neutralization, achieving wound reactivation. The primary aim of this study is to compare the effect of the antioxidant dressing on chronic wound healing against the use of a standard wound dressing in patients with hard-to-heal wounds.

**Methods:**

We will conduct a multicentre, single-blind, randomized controlled trial with parallel groups. Participants will be selected from three primary public health care centres located in Andalucía (southern Spain). Patients will be randomized into an intervention group (antioxidant dressing) or a control group (standard wound dressing). Assessments will be carried out at weeks 2, 4, 6 and 8. Follow-up will be for a period of 8 weeks or until complete healing if this occurs earlier.

**Discussion:**

The findings from this study should provide scientific evidence on the efficacy of the antioxidant dressing as an alternative for the treatment of chronic wounds. This study fills some of the gaps in the existing knowledge about patients with hard-to-heal wounds.

**Trial registration:**

ClinicalTrials.gov: NCT03934671. Registered on 2 May 2019.

## Background

A wound that does not heal in the orderly stages of the healing process or does not heal within 3 months is considered a chronic wound, also known as a hard-to-heal wound or non-healing wound [1]. Hard-to-heal wounds have been defined as any wound that has not healed by 40–50% after 4 weeks of appropriate treatment [2]. Chronic wounds are a major clinical and economic problem for health care institutions because of their impact on the quality of life of both patients and caregivers [3–5]. Wound healing is impaired when the wound remains in the inflammatory stage for too long [1]. A range of factors that can delay the healing process have been described, and include the imbalance between proteases and protease inhibitors in the wound bed [6–8], bacterial colonization and the presence of biofilm [9–11], and oxidative stress [1, 4, 5, 12].

Matrix metalloproteinases (MMPs) and their inhibitors contribute to the balance between extracellular matrix degradation and deposition, creating a balance that is essential for timely and coordinated healing [7, 8]. However, in chronic wounds, the inflammatory response is augmented, and this balance becomes disrupted. This situation is characterized by elevated levels of pro-inflammatory cytokines and MMPs. Therefore, wound dressings aimed at sequestering the MMPs within the wound milieu is one particular area of interest as an alternative wound management strategy [6].

Biofilm is associated with impaired epithelialization and granulation tissue formation, and promotes a low-grade inflammatory response that interferes with wound healing [9, 10]. Over 90% of chronic wounds have an aggregation of microorganism-forming biofilm [13]. Biofilm is a survival mechanism that confers to bacteria and other microorganisms the ability to resist environmental stressors and antimicrobials due to a variety of reasons, including low metabolic activity [11].

An excess of reactive oxygen species (ROS), including singlet oxygen, hydrogen peroxide and hydroxyl radicals, causes a pro-inflammatory environment in the wound bed [14–16], attracting more inflammatory cells into the wound and creating a negative feedback loop that could delay or prevent wound closure [1, 4, 5, 17, 18]. The strong oxidizing capabilities of ROS can therefore damage many of the molecules and structures of the cells [12]. It has been proposed that the use of a dressing that can exert an antioxidant effect on wound exudates might restore the appropriate ROS balance [19].

Recently, wound management has improved significantly. New materials and bioactive wound dressings have been developed that promote a favourable environment and promote active wound healing treatment, such as collagen dressings [20, 21], MMP modulator dressings [22], and dressings with antioxidant activity of inhibitors of free radical reactions [23, 24].

Among these new advanced products, there is an antioxidant dressing (Reoxcare®) that was developed by Histocell (Bizkaia, Spain). This dressing combines an absorbent matrix obtained from locust bean gum galactomannan with antioxidant properties [25] and a hydration solution with curcumin [26, 27] and N-acetylcysteine (NAC) [28, 29]. Curcumin is a natural phenol from the rhizome of the plant *Curcuma longa* that has been used for over 2000 years as an antioxidant and an anti-inflammatory, and specifically in wounds to improve healing [30–32]. NAC is widely applied as an antioxidant molecule, and has been recently successful for the treatment of wounds [28, 33]. These three components act as free radical scavengers since two of them also have a synergistic antioxidant effect [34]. Due to the innovative design, this antioxidant dressing combines the advantages of moist healing in exudate management and free radical neutralization, achieving wound reactivation. In addition, preliminary observations suggest that this antioxidant dressing may have antibiofilm activity to eliminate and prevent reformation [24]. These findings suggest that the dressing may represent a new advanced alternative for the management of hard-to-heal wounds.

This dressing with antioxidant properties has been tested in animal models and in case series of patients with acute wounds and chronic wounds of various aetiologies (venous ulcers, neuropathic, postsurgical), pressure ulcers and neuro-ischemic diabetic foot ulcers, showing favourable results in activating the healing process [33, 35]. Data have also been published that reflect an estimate of the cost benefit of treatment with antioxidant dressings in hard-to-heal wounds with venous vascular aetiology [36]. However, there is currently no study comparing this new antioxidant dressing with standard wound dressings that maintain a moist environment that are used in routine clinical practice for the treatment of chronic wounds. This trial aims to fill this gap in the knowledge.

### Hypotheses

The hypotheses for the trial are: 1) the use of the antioxidant dressing will reduce the percentage of nonviable tissue in the wound bed more than standard wound dressings; 2) the use of the antioxidant dressing will increase new granulation tissue formation with respect to standard wound dressings; and 3) the use of the antioxidant dressing will produce a higher rate of wound healing than standard wound dressings.

### Study objectives

The primary aim of this study is to compare the effect of an antioxidant dressing on the healing of chronic wounds against the use of dressings that create a moist environment (as standard clinical practice) in patients with hard-to-heal wounds.

The secondary aims are to measure the intrapatient variation over time in the percentage of nonviable and granulation tissue in the wound bed and to measure the reduction in the wound area. Variation in the area of the wound covered by nonviable tissue is important because its reduction is an early sign of the activation in the healing process (which is an expected effect of the new dressing).

## Methods

### Design

**Fig. 1:**
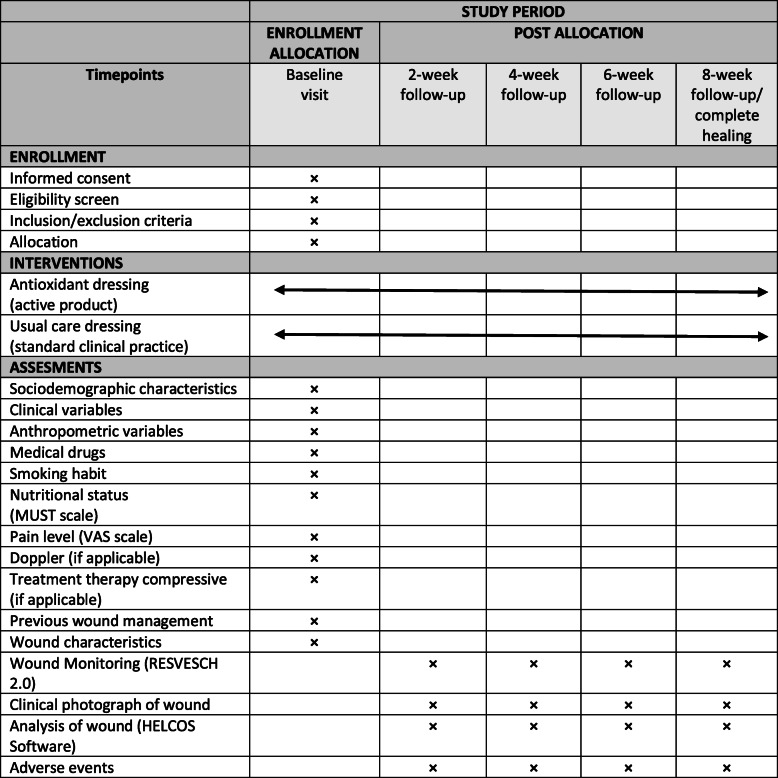
Standard Protocol Items: Recommendations for Interventional Trials (adapted from SPIRIT figure) schedule of enrolment, interventions and assessments. *MUST* malnutrition universal screening tool, *VAS* visual analogue scale

The REOX study is a multicentre, single-blind, randomized controlled trial with parallel groups. Figure 1 presents an overview of the schedule for enrolment, intervention and assessment according to the Standard Protocol Items: Recommendations for Interventional Trials (SPIRIT) guidelines (Additional file 1).

### Setting

The REOX study will run in three primary health care centres (one urban centre and two rural centres) of the Andalusian Health Service in Spain. The centres were selected taking into account that advanced practice wound nurses work in them who could participate in the study (detailed in Additional file 2).

#### Participants

Patients are eligible according the inclusion and exclusion criteria presented in Table 1*.* The sample was recruited from patients with wounds treated in one of the health care centres engaged in the study.

**Table 1:** REOX trial inclusion and exclusion criteria

Inclusion criteria	Exclusion criteria
• Patients over 18 years of age • Patients with leg ulcers (venous, ischaemic, traumatic or diabetic foot ulcer) • Patients with dehisced surgical wounds healing by second intention • Patients with pressure ulcers • Wound area between 1 and 250 cm^2^ (wounds larger than 250 cm^2^ are very unusual and outside the scope of this kind of treatment and therefore we decided to exclude it since such a large wound could be more difficult to heal)	• Systemic inflammatory disease or oncological disease • Wounds with clinical signs of infection • Terminal situation (life expectancy less than 6 months) • Ulcers from other aetiologies: tumours, infectious • Wounds treated with negative pressure therapy • Pregnancy • History of sensitivity or allergy to any of the components of the study dressing

In addition, the following criteria for withdrawal from the study will be considered: 1) worsening of the wound according to the clinical judgment of the professional (appearance of clinical signs of infection or others); 2) appearance of allergies or hypersensitivity to the dressing; 3) death; 4) hospital admission that interrupts the treatment in the primary health care centre; and 5) change in the patient’s residence if the new residence is at a different health centre.

#### Sampling

Sample size was estimated based on two possible outcomes. First, to detect a difference of 2 points in the RESVECH 2.0 scale mean scores [37] between the intervention and the control group with a standard deviation of 2.0 (5% type 1 error and 20% type 2 error) based on data published by Castro et al. who found a reduction of 8 points in RESVECH 2.0 at 4 weeks of treatment (this is a 23% reduction) [24]. We used a 2-point reduction for sample estimation as the minimum clinically significant difference, and so the study was powered enough to detect just this small difference. The required sample size for this is 17 patients per group. Second, the time to achieve a 50% reduction of the wound area, estimating an average of 49.5 days in the control group and 30.0 days in the intervention group with a standard deviation of 20.0 days (5% type 1 error and 20% type 2 error), as based on the study by Lee [38]. The required sample size for this is 18 patients per group.

An additional 30% is added to compensate possible loss of patients in follow-up, and so the final sample size is 54 patients (27 patients in each group). It is expected that each one of the three health care centres will recruit 18 patients for the study.

### Randomization and blinding

**Fig. 2:**
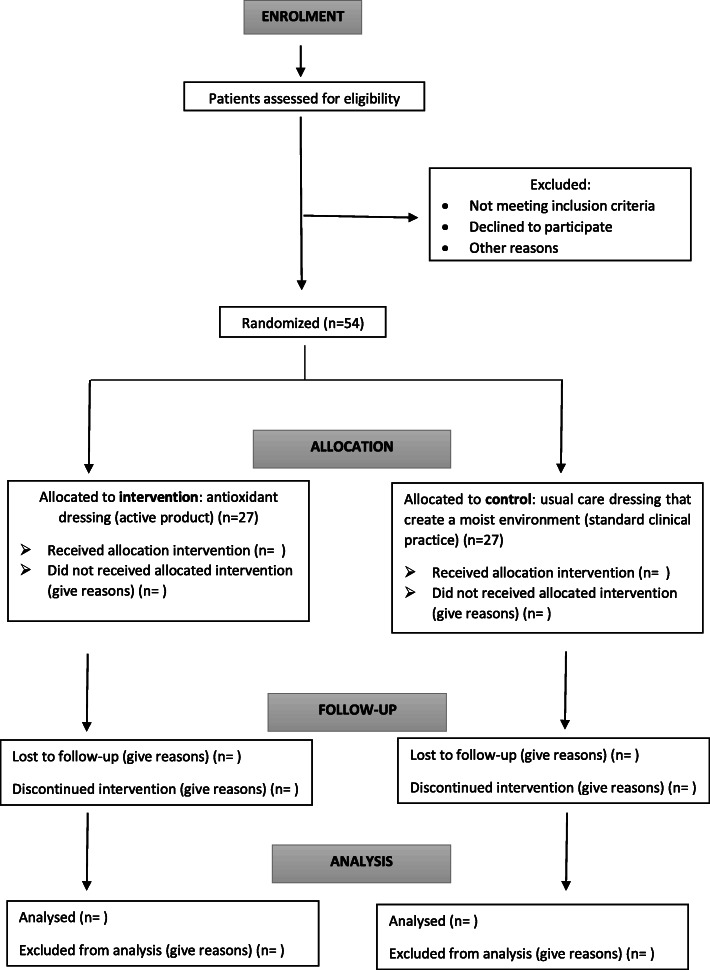
Schedule of enrolment

Patients (*n* = 54) will be randomized into an intervention group (antioxidant dressing) or control group (standard clinical practice using dressings that create a moist environment). The block randomization scheme was generated using the EPIDAT software. Participants will be randomized to receive antioxidant dressing or standard wound dressing with a 1:1 allocation ratio (Fig. 2) using stratified block randomization with a fixed block size per centre. Once the patient has agreed to participate and has been shown to meet the inclusion criteria, the clinical nurse will open opaque closed envelopes sequentially numbered containing a sheet (folded four times) with the group allocation. Neither the patient nor the clinical nurse is blinded to the treatment received; the data and the wound pictures will be codified and the assessor who evaluates the wound pictures and the data analyst will be blinded to the treatment.

### Intervention and control

The REOX study has two arms (see Fig. 2). Both arms included cleansing with saline solution and debridement to remove devitalized tissues, if needed, according to recommendations from clinical practice guidelines.

In the intervention group, the antioxidant dressing will be applied over the wound bed and covered with a secondary adhesive dressing. The dressing will be kept in place for 2 to 3 days, according to the recommendations of the manufacturer.

In the control group (standard practice), a dressing that creates a moist environment will be applied over the wound bed (such as hydrocolloid, alginate, polyurethane foam or silver dressing, according to the nurse’s judgement and the availability of the products at the centre) and covered with a secondary adhesive dressing if necessary. The dressing will be kept in place for 2 to 3 days, according to the recommendations of the manufacturer.

To improve the adherence of the patients to the treatment (both groups), the clinical nurse who treats the patients will reinforce in each dressing change how important it is to keep the dressing in place until the next visit, and explain to the patient that if they are experiencing pain, itching or other wound-related symptoms, they should consult with the nurse but not remove the dressing. Any dressing-related symptoms will be recorded as a possible adverse effect.

### Data collection and management

Participants eligible for study entry will be given a unique, sequential, centre-specific study identifier. Clinical data will be entered into paper-based case report forms. After each assessment, the data will be handled according to internal procedures and stored in a secure server. These files will be backed-up into a password-protected environment. Hard copies will be stored locally, in compliance with the Spanish Data Protection Law (2018).

Each patient included in the study will be assessed by one clinical nurse at baseline and every 2 weeks (weeks 2, 4, 6 and 8). Figure 1 shows the variables collected at each assessment. The follow-up of patients in both groups will be continued until week 8 or until complete healing of the wound if this occurs earlier. A follow-up of 8 weeks was considered adequate for this study since the main action of the antioxidant dressing being evaluated is to reactivate the healing process by avoiding the excess of ROS; according to a previous study published by Castro et al. [35], a significant number of wounds improved by the fourth week. In order to promote participant retention and complete follow-up, the nurse will monitor patients in the health care centre. Furthermore, the principal investigator will have the primary responsibility for study monitoring; a formal data monitoring committee is not considered necessary because there is no access to protected information and there is no personal information recorded.

To monitor wound healing, several variables will be used: 1) wound area by direct measurement of length and width with a graduated ruler (Kundin method) [39]; 2) wound area estimated using digital photography with Helcos software [40, 41]; this software is an integrated wound management system, which allows for estimation of the wound area and the relative percentage of tissue types in the wound bed (granulation, slough and necrotic tissue); and 3) the RESVECH 2.0 scale score for chronic wound healing [37] (this scale assesses six aspects — wound size, depth/affected tissues, wound edges, type of tissue in the wound bed, exudate and infection/inflammation — with a higher score showing more improvement in the healing process from 0 (the worst possible status of the wound) to 35 points (wound healed)).

Other variables that will be recorded are demographic characteristics, anthropometric characteristics, pain level, concomitant medical diagnosis and clinical antecedents, nutritional status, consumption of drugs, tobacco habit, ankle–brachial index and treatment with compressive therapy. No biological specimens will be collected or stored in this study.

### Outcomes

The primary outcome is the improvement in wound healing with two end points: 1) an increase in the RESVECH 2.0 scale score [37] (score at baseline minus score at 8 weeks); and 2) a reduction in the wound area (area at baseline minus area at 8 weeks). The RESVECH 2.0 score includes the assessment of the wound area, but we also decided to use the reduction in wound area as a specific end point to have a direct measurement of wound size (despite not estimating the sample size with this outcome because there were no data available from previous studies). Additionally, the values at 2, 4 and 6 weeks for each end point will be calculated. These end points are chosen for the clinical relevance of the antioxidant dressing because this product is well-known for removing the nonviable tissue (necrotic or devitalized) in the wound bed, for promoting the production of granulation tissue and for activation of the healing process [35, 42].

The secondary outcomes are the time to achieve a 50% reduction in wound area, the time elapsed until removal of nonviable tissue from the wound bed, reduction of the wound area, the number of completely healed wounds, the pain level and the area of the wound with bacterial load (clinical signs of infection or measurement of surfaces containing bacteria).

### Data analysis plan

A researcher who is blinded to group allocation will perform the data analysis. All analyses will be performed with SPPS Statistics software (IBM Corp., Armonk, NY, USA). Data will be expressed as frequency and percentages for categorical variables, and mean, standard deviation, range and median for quantitative variables depending on the distribution (normality will be checked with the Shapiro–Wilk test). The statistical significance level will be set at *P* < 0.05.

For the primary outcome of improvement in wound healing the wound area reduction at 8 weeks in the intervention group compared with the standard care group will be tested by a difference of means test. The increase in RESVECH 2.0 [37] score at 8 weeks between both groups will be examined by a difference of means test (*t* test for independent groups or Mann–Whitney test depending if variables adjust to a normal distribution). The effect size will be estimated by Cohen’s *d* statistic. In addition, a repeated-measures analysis (analysis of variance or Friedman test) and a survival analysis (Kaplan–Meier) will be used to analyse the evolution over time.

For secondary outcomes, proportions and categorical variables will be tested using a chi-squared test, and continuous variables will be tested using a mean difference test (parametric or nonparametric).

Analyses will be performed between groups as randomized (an intention-to-treat analysis). Missing data for the outcomes will be tested if they are missing at random; otherwise the last observation carried forward approach will be used for imputation [43]. Furthermore, sensitivity analyses over all the completed cases (a per-protocol analysis) will be performed to check for inconsistencies.

A subgroup analysis by wound type will also be performed to explore whether the effect of the intervention differs for any type of wound according to its aetiology. Moreover, another subgroup analysis by primary care centre will be carried out to check if there are any differences in outcomes according to the centre.

No interim analyses or formal stop-study rules are planned; no harmful problems are anticipated for patients because this new dressing has been previously tested in a series of patients with wounds of various aetiologies without any serious adverse effects [35].

### Oversight and monitoring

The project team at the University of Jaén is responsible for the design, preparation and coordination of the trial, including the dissemination of the results. This team meets every month to discuss the progress of the trial and to solve any arising problems. A steering committee, formed by two professors from the Nursing Department of the University of Jaén and a member of Histocell, will supervise the trial. This committee will meet twice a year during the course of the study and when the data collection is complete. At each primary care health centre participating in the study a clinical nurse will be responsible for recruiting patients, obtaining informed consent and coordinating the trial at the site. In addition, some site visits are planned every 3–4 months to audit the trial and monitor compliance with the study design. Regular reports with data on recruitment and follow-up rates will be sent to the principal researcher every month. Any deviation from the protocol will be fully documented by a breach report. Any significant amendments to the protocol will be communicated to the relevant parties and updated in the trial register.

### Dissemination plan

The findings obtained in this study will be disseminated at conferences about wound management (mainly at European level) and other relevant national and international meetings. The key findings will be reported early by social media and the website of the research group, and will be updated in the trial register. The full study report will be submitted for publication in a specialist wound journal, preferably an open-access journal.

## Discussion

This randomized clinical trial will be the first to compare the effect on chronic wound healing using an antioxidant dressing with the use of a dressing that creates a moist environment (standard care) in patients with hard-to-heal wounds.

Currently, through the research of scientists, engineers and manufacturers, we are witnessing an increase in scientific knowledge about wound healing; this is associated with the development of products that promote positive results and mitigate negative factors in wound healing. All of these research findings and resources are transferred to clinical practice through evidence-based wound management [44].

The dressing with antioxidant properties has been previously tested in animal models [33] and in patients with acute and chronic wounds of various aetiologies, showing a potential to activate the healing process [35]. The antioxidant effect of NAC may play a role for tissue repair under normal and pathological conditions, thus improving wound healing [28, 42].

In addition, some recently published data point to a positive cost–benefit balance for the treatment of venous ulcers with an antioxidant dressing [24, 36].

A potential limitation of this study may arise because of the inclusion of wounds with different aetiologies, so any imbalance in the allocation could confound the results. This is important for diabetic foot ulcers and for venous ulcers. This limitation will be managed during the study through balancing the type of wounds included and through subgroup analysis (after data collection).

Thus far, no study has been conducted comparing this new antioxidant dressing with standard dressings to maintain a moist environment. If our results confirm the hypothesis, this randomized clinical trial could help provide new clinical evidence of the efficacy of the antioxidant dressing as an alternative for the treatment of chronic wounds. This study aims to fill some of the gaps in the existing knowledge of advanced therapies for chronic wounds.

## Trial status

The study is registered on ClinicalTrials.gov (NCT03934671), https://clinicaltrials.gov/ct2/show/NCT03934671, registered on 2 May 2019. The Ethics Committee of Jaén approved the study in April 2019 with reference number 0645-N-19. This protocol is version 2.0, April 2020. Recruitment began in September 2019. The expected date for recruitment completion is December 2020.

## Supplementary information

**Additional file 1.** SPIRIT 2013 checklist: recommended items to address in a clinical trial protocol and related documents.

**Additional file 2.** Collaborating primary health care centres in the REOX study, and expected number of patients recruited.

## Data Availability

The data generated in this study can be shared after reasonable request to the corresponding author.

## Abbreviations

MMP: Matrix metalloproteinase
NAC: N-acetylcysteine
ROS: Reactive oxygen species
