# Supplementary material for: Antioxidant dressing therapy versus standard wound care in chronic wounds (the REOX study): study protocol for a randomized controlled trial
Source: Trials. 2020 Jun 8;21:505. doi: 10.1186/s13063-020-04445-5 (PMC7278054; doi:10.1186/s13063-020-04445-5)
Supplement: Supplementary file 2 — Additional file 2. Collaborating primary health care centres in the REOX study, and expected number of patients recruited. [file 13063_2020_4445_MOESM2_ESM.docx]

| **Primary health care centres** | **Number of patients expected to be recruited (n)** |
| --- | --- |
| Health Care Centre (Jaén) | 18 |
| Health Care Centre (Almería) | 18 |
| Health Care Centre (Málaga) | 18 |

**Additional File 2**. Collaborating primary health care centres in the REOX study, and expected number of patients recruited.
